# The role of ethanolamine phosphate phospholyase in regulation of astrocyte lipid homeostasis

**DOI:** 10.1016/j.jbc.2021.100830

**Published:** 2021-05-26

**Authors:** Cory J. White, Jessica M. Ellis, Michael J. Wolfgang

**Affiliations:** 1 Department of Biological Chemistry, The Johns Hopkins University School of Medicine, Baltimore, Maryland, USA; 2 Department of Physiology, East Carolina Diabetes and Obesity Institute, East Carolina University, Greenville, North Carolina, USA; 3 Department of Pharmacology and Molecular Sciences, The Johns Hopkins University School of Medicine, Baltimore, Maryland, USA

**Keywords:** phosphatidylethanolamine, astrocyte, liver, glucocorticoid, lipid, brain, AA, arachidonic acid, ANOVA, analysis of variance, CNS, central nervous system, DHA, docosahexaenoic acid, *eas*, easily shocked, EtN, ethanolamine, Etnppl, ethanolamine phosphate phospholyase, FAO, fatty acid oxidation, HBSS, Hank’s buffered salt solution, HSP, hereditary spastic paraplegia, KO, knockout, PC, phosphatidylcholine, PE, phosphatidylethanolamine, PEtN, phosphoethanolamine, PFC, prefrontal cortex, PUFA, polyunsaturated fatty acid, qRT-PCR, quantitative real-time polymerase chain reaction, TRAP, translating ribosomal affinity purification

## Abstract

Dietary lipid composition has been shown to impact brain morphology, brain development, and neurologic function. However, how diet uniquely regulates brain lipid homeostasis compared with lipid homeostasis in peripheral tissues remains largely uncharacterized. To evaluate the lipid response to dietary changes in the brain, we assessed actively translating mRNAs in astrocytes and neurons across multiple diets. From this data, ethanolamine phosphate phospholyase (*Etnppl*) was identified as an astrocyte-specific fasting-induced gene. Etnppl catabolizes phosphoethanolamine (PEtN), a prominent headgroup precursor in phosphatidylethanolamine (PE) also found in other classes of neurologically relevant lipid species. Altered *Etnppl* expression has also previously been associated with humans with mood disorders. We evaluated the relevance of Etnppl in maintaining brain lipid homeostasis by characterizing Etnppl across development and in coregulation with PEtN-relevant genes, as well as determining the impact to the brain lipidome after *Etnppl* loss. We found that *Etnppl* expression dramatically increased during a critical window of early brain development in mice and was also induced by glucocorticoids. Using a constitutive knockout of Etnppl (Etnppl^KO^), we did not observe robust changes in expression of PEtN-related genes. However, loss of Etnppl altered the phospholipid profile in the brain, resulting in increased total abundance of PE and in polyunsaturated fatty acids within PE and phosphatidylcholine species in the brain. Together, these data suggest that brain phospholipids are regulated by the phospholyase action of the enzyme Etnppl, which is induced by dietary fasting in astrocytes.

The structure, function, and biochemical mechanisms of the brain can be affected by the lipid composition of one’s diet and respective metabolic state. However, how metabolic cues impact lipid homeostasis and neurologic function is not well understood. For example, long-chain polyunsaturated fatty acids (PUFAs) such as docosahexaenoic acid (DHA) are enriched in the brain and are critical for neurologic development, but are also essential nutrients dependent on dietary intake in humans and rodents (1, 2, 3, 4, 5). Furthermore, neurologic dysfunctions such as depression (6) and autism spectrum disorders (7, 8, 9, 10, 11) have been associated with altered lipid metabolism. People with genetic disorders in lipid metabolism can also suffer from neurologic impairments including encephalopathies, seizures, and cortical atrophy (12, 13, 14). In humans, genetic errors in metabolism of phospholipids, sphingolipids, and other complex lipids such as triacylglycerols can result in a number of neurologic symptoms such as but not limited to spasticity and weakness of the lower extremities, dementia, loss of vision, ataxia, epilepsy, encephalopathy, and lipid accumulations in the brain tissue in disorders such as hereditary spastic paraplegia (HSP) (*via* loss of ECT, EPT1, or DDHD2) (15, 16, 17), Sjogren-Larsson syndrome (*via* loss of ALDH3A2) (18, 19), and Chanarin–Dorfman syndrome (*via* loss of ABHD5) (18, 20). While it is clear that the unique lipid biochemistry of the nervous system is important for function, the regulation of lipid metabolism in the CNS is not well understood.

Ethanolamine phosphate phospholyase (*Etnppl*, formerly known as *Agxt2l1*) is a gene that encodes for Etnppl protein, which was recently characterized to irreversibly degrade phosphoethanolamine (PEtN) to acetaldehyde, ammonium, and inorganic phosphate with high specificity (21, 22, 23, 24, 25). *Etnppl* was identified based on homology to aminotransferase-like phosphorylases, and its catalytic activity was characterized using purified Etnppl protein *in vitro* (24). PEtN is a small amino acid that is involved in the metabolic pathways of several types of lipids. Most notably, PEtN is necessary to generate the phospholipid phosphatidylethanolamine (PE) *via* the Kennedy pathway (also known as the CDP-ethanolamine pathway) (26, 27). PE is the primary inner leaflet phospholipid found in membrane phospholipid bilayers (28). Additionally, PEtN is also found as a by-product of PE, sphingosine, endocannabinoid, and plasmalogen breakdown as well as being a potential inhibitor of mitochondrial respiration (29).

Furthermore, PEtN-related metabolic pathways and PEtN abundance have been observed to have implications on neurologic function when altered. One of the first clues that phospholipid metabolism directly affects neural metabolism was found in the *Drosophila* mutant easily shocked (*eas*). The mutant, *eas*, maintains normal behavior under most conditions but after a brief mechanical shock, exhibits seizure and paralysis (30). This gene was mapped and identified as the ethanolamine kinase gene of the Kennedy pathway, which is responsible for generating PEtN. These mutant flies are unable to generate the critical intermediate PEtN and have an altered membrane lipid composition. These neurologic deficits can be rescued in *Drosophila* by re-expression of *eas* in adults (31). Therefore, regulating PEtN is important for proper nervous system function.

Etnppl protein is primarily expressed in the brain (24, 32, 33), specifically astrocytes in the central nervous system (CNS), and liver (24), and it has been found to have altered gene expression in a number of neurologic and hepatic impairments. For instance, in the brain, there is a twofold increase in *Etnppl* mRNA in postmortem prefrontal cortices (PFCs) from patients who suffered from common neurologic disorders including schizophrenia or bipolar disorder (34). Conversely, *Etnppl* mRNA is down 72% in PFCs from depressed patients (35). Interestingly, *Etnppl* mRNA is upregulated in mice treated with HDAC inhibitors or lithium; the latter commonly used to treat bipolar disorder (36, 37). Furthermore, ETNPPL protein has been found to have decreased expression in human glioma brain tumors, which further decreases with malignant progression (38). Knockdown of Etnppl protein in human hepatocellular carcinoma and cholangiocellular carcinoma tissues perturbs lipid synthesis and hypothesized to modulate lipogenesis based upon gene set enrichment analysis (39).

We identified Etnppl, a phospholipid-precursor catabolizing gene as a potentially important contributor to brain lipid metabolism homeostasis because it was upregulated specifically in astrocytes after dietary fasting. In Etnppl induction, we have determined a novel enzyme in the brain regulated by metabolic state. To better understand the biological function of Etnppl, which is largely uncharacterized, we utilized constitutive Etnppl knockout (KO) mice (ETNPPL^KO^) to examine the changes in Etnppl and other PEtN-related genes across development, across diets, and the impact of Etnppl loss on the brain lipidome to understand its contributions particularly to the regulation of brain phospholipids.

## Results

### *Etnppl* is regulated by fasting in CNS astrocytes

Neurological disorders, including epileptic seizures, often exhibit improved outcomes with fasting or ketogenic diets. However, how altered metabolic cues, such as a dietary change, impact gene expression and therefore lipid homeostasis to improve neurologic function within the CNS is not well understood. To assess how gene expression in the brain is altered *in situ* by nutritional cues, we performed translating ribosomal affinity purification (TRAP) (40) in several cell types in the CNS upon nutritional modulation. TRAP mice express a ribosomal epitope tag upon Cre-induced recombination that can be immunoprecipitated. We measured the abundance of actively translating mRNAs from a ribosomal pull-down that came from adult astrocyte (Aldh1l1-Cre)- or neuron (Syn1-Cre)-specific TRAP mice that were subjected to one of three dietary conditions: 4 weeks of normal chow diet, 4 weeks of ketogenic diet (high-fat, low-carbohydrate) (41), or an 18-h fast. Immediately following the respective diets, the forebrain and hippocampus were harvested from all groups, ribosomes were immunoprecipitated, and actively translating mRNAs in the ribosomes were purified. Samples from astrocyte-specific TRAP mice were further used for whole exome microarray analysis (Fig. S1).

**Figure 1:**
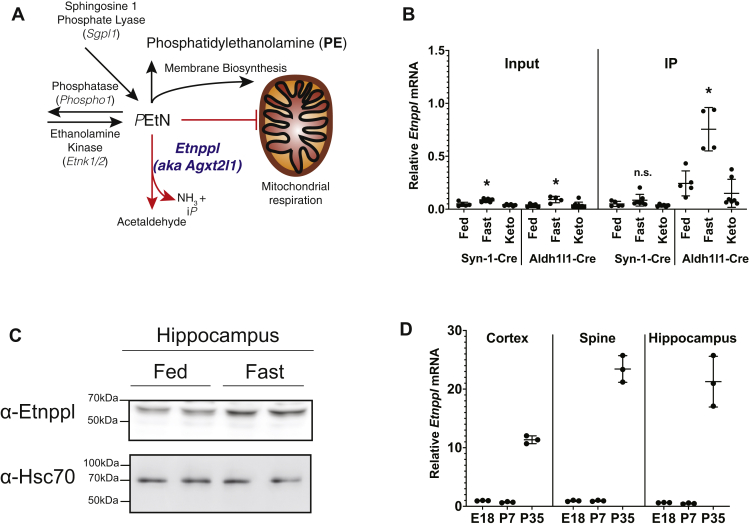
**Identifying dietary regulation of genes in neurons and astrocytes using translating ribosomal affinity purification (TRAP).***A*, graphic showing possible metabolic outcomes of phosphoethanolamine (PEtN). *B*, *Etnppl* mRNA expression using qRT-PCR of input and pulled-down (IP) neuron (Syn-Cre) and astrocyte (Aldh1l1-Cre) fractions tissue harvested from ribo-tag mice exposed to fed, fasted diet, or ketogenic diet of TRAP screen. Samples sizes [Syn-Cre: fed (n = 5), fasted (n = 7), ketogenic diet (n = 6)]. Aldh1l1-Cre: fed (n = 5), fasted (n = 4), ketogenic diet (n = 8). *C*, protein expression of Etnppl from fed and overnight fasted adult mice n = 3. *D*, *Etnppl* mRNA expression using qRT-PCR across ages (prenatal day 18 (E18) and postnatal day 7 and 35 (P7 and P35) and CNS regions (cortex, spine, and hippocampus)). (n = 3) Data are expressed as mean ± S.D. Represented data analyzed using multiple Student’s two-tailed t-tests. ∗α = 0.05; ∗∗α = 0.01; ∗∗∗α = 0.001; ∗∗∗∗α = 0.0001; ns, not significant.

Microarray analysis from forebrain astrocytes showed that ethanolamine phosphate phospholyase (*Etnppl*) was elevated 11.4-fold in the forebrain astrocytes derived from fasted mice. Etnppl irreversibly degrades the PE intermediate PEtN to acetaldehyde, ammonia, and inorganic phosphate and has been implicated in multiple psychosocial disorders (22, 23, 24). Thus, modulation of *Etnppl* expression and/or activity could potentially impact cognitive function *via* phospholipid homeostasis. The PE intermediate PEtN is at the intersection of several lipid pathways including the biosynthesis of PE (27), a breakdown product of sphingosine-1-phosphate (42), endocannabinoids (43, 44), or plasmalogen (16), and has been implicated as an inhibitor of mitochondrial respiration (29) (Fig. 1*A*). Therefore, we selected Etnppl for further analysis.

The induction of *Etnppl* in astrocytes upon fasting was validated using quantitative real-time polymerase chain reaction (qRT-PCR) with hippocampal astrocyte- and neuron-specific samples across chow, ketogenic, and fasted diets from input (total RNA) and immunoprecipitated fractions from TRAP mice. *Etnppl* was induced threefold by fasting in total (input) mRNA compared with fed samples (Fig. 1*B*). Also, we confirmed that the induction of *Etnppl* is astrocyte-specific with no induction in neuron-specific IPs (Fig. 1*B*). Consistent with these observations, hippocampal Etnppl protein, determined using a C-terminal directed Etnppl antibody developed by our laboratory, was induced by fasting (Fig. 1*C*). These data show that *Etnppl* is induced by fasting in CNS hippocampal astrocytes.

### *Etnppl* and ethanolamine metabolic enzymes are developmentally regulated

Next, we determined *Etnppl* mRNA expression across tissues in fed and overnight fasted adult mice using qRT-PCR. *Etnppl* is highly expressed in the brain and liver with expression also found in the kidney and gut (Fig. S2). When examining the mRNA expression across regions of the brain including cortex, hippocampus, and cerebellum, we observed that *Etnppl* was highly developmentally regulated postnatally (Fig. 1*D*).

**Figure 2:**
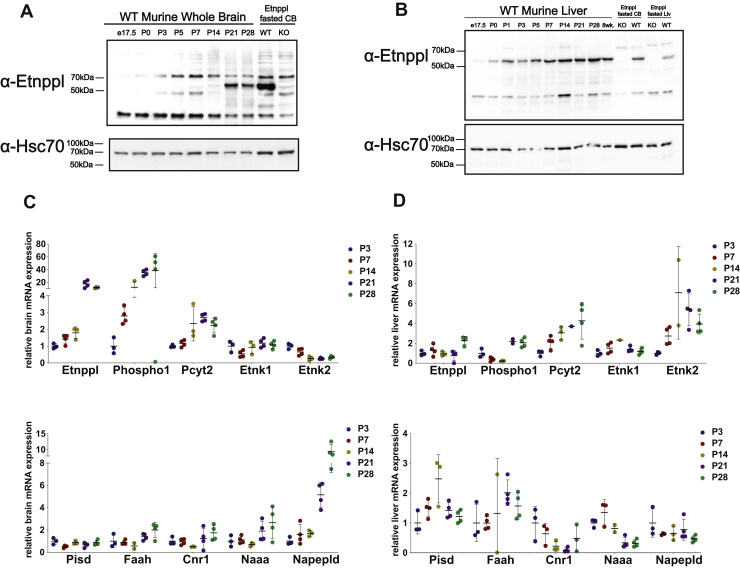
**Expression of Etnppl and PEtN-related genes across early development.***A*, Etnppl protein expression in the whole brain across ages. WT and Etnppl^KO^ fasted cerebellum (CB) tissue used as positive and negative controls for Etnppl protein expression repectively. *B*, Etnppl protein expression in the liver across ages. WT and Etnppl^KO^ fasted CB and liver tissue used as positive and negative controls for Etnppl protein expression repectively. *C*, *Etnppl* and PEtN-related gene mRNA expression in the whole brain using qRT-PCR across ages P3 (n = 3), P4 (n = 4), P14 (n = 3), P21 (n = 4), P28 (n = 4). *D*, *Etnppl* and PEtN-related gene mRNA expression in the liver using qRT-PCR across ages P3 (n = 3), P4 (n = 4), P14 (n = 3), P21 (n = 4), P28 (n = 4). Data (as mentioned in text) are expressed as mean ± S.D. Represented data analyzed using ordinary measures two-way analysis of variance with Sidak’s tests for multiple comparisons. Outliers were removed after using Grubb’s outlier test. ∗α = 0.05; ∗∗α = 0.01; ∗∗∗α = 0.001; ∗∗∗∗α = 0.0001; ns, not significant.

To better understand the developmental regulation of *Etnppl*, we further characterized its expression within the window of the most dramatic increase of *Etnppl* expression (between P7 and P35). We collected the brain and liver from chow-fed mice for determination of Etnppl protein expression at the following ages: P3, P7, P14, P21, and P28. In the brain, a lack of Etnppl protein expression was evident as late as P14 followed by a stark increase in expression between P14 and P21 (32, 45) (Fig. 2*A*). In the liver, expression increases earlier in development and more steadily throughout the duration of the developmental window (Fig. 2*B*).

To determine if *Etnppl* expression is coregulated with the expression of other PEtN-related genes, we performed qRT-PCR using the whole brain and liver from the same ages of mice as above. PEtN-related genes that were investigated included the PEtN phosphatase *Phospho1*, PE-biosynthetic Kennedy pathway genes (*Etnk1*, *Etnk2 Pcyt2*), endocannabinoid genes (*Faah*, *Cnr1*, *Naaa*, *Nape-Pld*), and the PE-biosynthetic gene *Pisd*. In the whole brain, *Etnppl* mRNA expression over the course of development followed similar expression patterns as observed with Etnppl protein. *Etnppl* mRNA increased a dramatic 9.8-fold between P14 and P21 (Fig. 2*C*). *Phospho1* followed a similar developmental trend as *Etnppl as* mRNA expression increased starkly 4.4-fold between P7 and P14 (Fig. 2*C*). The PE-biosynthetic Kennedy pathway gene *Pcyt2* increases steadily 2.70-fold between P3 and P21 (Fig. 2*C*). The Kennedy pathway gene ethanolamine kinase 2 (*Etnk2*) interestingly decreases significantly 2.46-fold between P7 and P14 while the isoform *Etnk1* is expressed uniformly at all ages (Fig. 2*C*). These data show that *Etnppl* and other ethanolamine metabolizing enzymes are highly regulated postnatally in the CNS.

Endocannabinoid-related genes are also coregulated throughout development in a similar trend as *Etnppl* in the whole brain (Fig. 2*C*). Most notably, *Napepld* mRNA expression, *Napepld* catalyzes the hydrolysis of N-acylethanolamines from N-acyl-PEs and is important for biogenesis of several endocannabinoids (46), is significantly increased threefold between P14 and P21, and further increased 5.5-fold at P28 (compared with P14) (Fig. 2*C*). Other endocannabinoid-related genes have increased mRNA expression at this same developmental time point between P7 and P14 but none as robustly as *Napepld*. The amide hydrolases *Faah* and *Naaa*, which are responsible for catabolizing the prominent endocannabinoid anandamide and palmitoylethanolamide respectively (46), have trending but not significant increases when comparing P14 with P21. *Faah* was increased 2.5-fold and *Naah* 2.7-fold, respectively (Fig. 2*C*).

In the liver, *Etnppl* mRNA expression was more similar across ages with a marked increase at P28 in comparison to the whole brain *Etnppl* mRNA and even when compared with the liver Etnppl protein expression (Fig. 2, *B* and *D*). When looking at coregulation of mRNA expression of PEtN-related genes across development in fed wild-type liver, no genes displayed the robust changes as seen in the whole brain, but some expression patterns were conserved. Specifically, *Phospho1*, *Pcyt2*, and *Etnk2* increase over the course of development (Fig. 2*D*). Expression of most other genes including *Pisd*, *Faah*, *Cnr1*, *Napepld*, *Etnk2* is variable across development while genes such as *Etnk1* remain consistent throughout development (Fig. 2*D*). These data show that EtN-related metabolic genes are upregulated in the CNS in a postnatal manner.

### Coregulation of PEtN-related genes across diets

**Figure 3:**
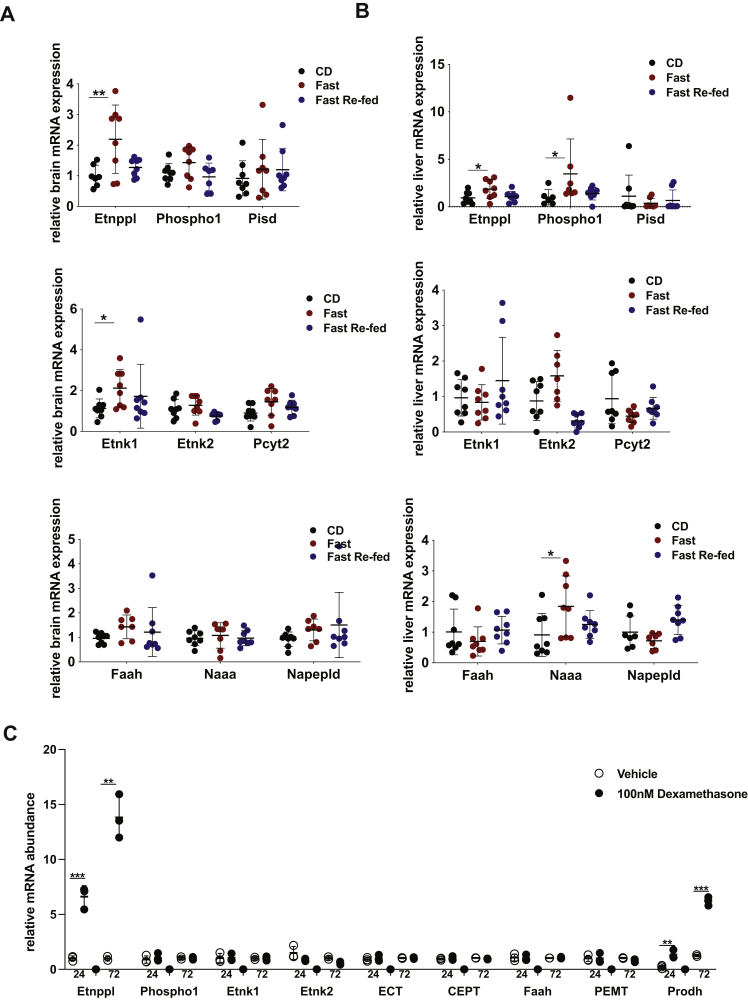
**Examining regulation of PEtN-related genes by diet and glucocorticoids.***A*, mRNA expression of Etnppl and other PEtN-related genes in the whole brain from adult chow-diet-fed, fasted, and refed after fasted mice using qRT-PCR. (n = 8). *B*, mRNA expression of Etnppl and other PEtN-related genes in the liver from adult chow-diet-fed, fasted, and refed after fasted mice using qRT-PCR. (n = 8). *C*, mRNA expression of PEtN-related genes in wild-type P2 1° astrocytes after a 24- or 72-h exposure to the glucocorticoid agonist dexamethasone. [dexamethasone] = 100 nM. (n = 3). Data in *A* and *B* are expressed as mean ± S.E.M. Represented data analyzed using ordinary measures two-way analysis of variance with Sidak’s tests for multiple comparisons. Data in *C* is expressed as mean ± S.D. Represented data analyzed using Student’s two-tailed t-tests. ∗α = 0.05; ∗∗α = 0.01; ∗∗∗α = 0.001; ∗∗∗∗α = 0.0001; ns, not significant.

*Etnppl* mRNA expression is induced by fasting (Fig. 1, *B* and *C*). Given that other relevant PEtN-related genes are developmentally coregulated, we determined if they may also respond to similar metabolic cues. Since Etnppl, *via* regulation of PEtN, intersects with several pathways of lipid metabolism, a better understanding of how diet impacts expression of these PEtN-related enzymes may provide insight to the functional role(s) of Etnppl (24, 29, 42, 43, 44, 47). To accomplish this, we collected the whole brain and liver samples from adult male mice subjected to one of the following before tissue harvest: a normal chow diet, an overnight 18-h fast, or refeeding following the overnight fast. In the brain, several PEtN-related genes, in addition to *Etnppl* (elevated 2.2-fold), were significantly increased after the overnight 18-h fast (Fig. 3*A*). Genes that were induced included PEtN kinase *Etnk1*, which was elevated 1.9-fold (Fig. 3*A*). Overall, the brain mRNA expression in refed animals was most similar to chow-diet-fed animals. In the liver, mRNA expression across diets varied widely from that in the brain (Fig. 3*B*). Other than *Etnppl* (increased significantly twofold), *Phospho1* and *Naaa* mRNA expressions in the liver are both significantly elevated by an overnight fast 3.5-fold and twofold, respectively (Fig. 3*B*). These data suggest that many other PEtN-related genes follow similar expression patterns due to changes in diet as Etnppl suggesting that Etnppl itself modulates the brain lipid metabolism homeostasis.

### *Etnppl* is induced by activation of the glucocorticoid receptor in astrocytes

*Etnppl* mRNA expression was shown to increase approximately twofold in mice introduced to an acute stressor (48). We reasoned that the rise in *Etnppl* mRNA expression by fasting may be due to glucocorticoids (49). To determine if PEtN-related gene expression is altered by activation of the glucocorticoid receptor, we measured mRNA expression of a panel of PEtN-related genes in wild-type, postnatal day 2 (P2) mouse primary (1°) astrocytes after either a 24-h or 72-h exposure to 100 nM of the glucocorticoid agonist dexamethasone or vehicle control *via* qRT-PCR (Fig. 3*C*). As a positive control, known glucocorticoid receptor responsive genes including *Prodh*, *Fkp5*, *Klf9*, *Sgk1*, *Mertk*, *Folh1*, *Gjb6*, and *Ch25h* were all induced by 100 nM dexamethasone at either one or both time points (Fig. S3). *Etnppl* mRNA was dramatically induced by dexamethasone, at 24-h and 72-h incubations, 6.6-fold and 13.8-fold, respectively (Fig. 3*C*). When examining other PEtN-related genes, few significant changes occurred due to dexamethasone exposure in 1° astrocytes (50). These data show that *Etnppl* is regulated by glucocorticoids in astrocytes.

### Loss of Etnppl has a minimal impact on PEtN-related gene expression

**Figure 4:**
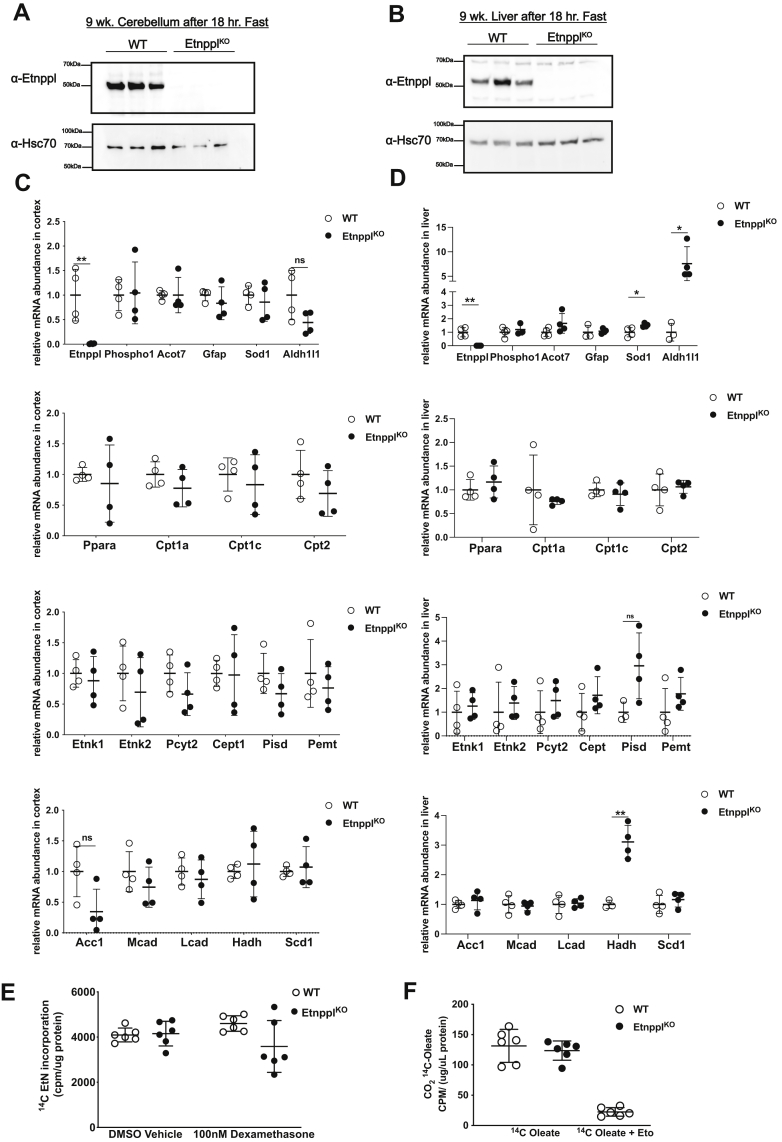
**Impact of Etnppl loss on gene expression and metabolic substrate use in the brain and liver.***A*, Etnppl protein expression in cerebellum from 9-week-old, 18-h fasted Etnppl ^KO^ and WT mice. *B*, Etnppl protein expression in liver from 9-week-old, 18-h fasted Etnppl ^KO^ and WT mice. *C*, cortex mRNA expression of *Etnppl*, genes that are indicators of CNS health, PEtN-related genes, and other metabolically relevant genes from 9-week-old, 18-h fasted Etnppl^KO^ and WT mice. n = 4. *D*, liver mRNA expression of *Etnppl*, genes that are indicators of CNS health, PEtN-related genes, and other metabolically relevant genes from 9-week-old, 18-h fasted Etnppl^KO^ and WT mice. n = 4. *E*, oxidation of 1-^14^C oleic acid to ^14^CO_2_ in P2 1° cortical astrocytes derived from Etnppl^KO^ and WT mice (n = 6). [etomoxir] = 100 μM. *F*, incorporation of 1-^14^C ethanolamine into membranes in cultured P2 1° cortical astrocytes derived from Etnppl^KO^ and WT mice (n = 6). [dexamethasone] = 100 nM. Data are expressed as mean ± S.D. Represented data analyzed using Student’s two-tailed t-tests. Outliers were removed after using Grubb’s outlier test. ∗α = 0.05; ∗∗α = 0.01; ∗∗∗α = 0.001; ∗∗∗∗α = 0.0001; ns, not significant.

We obtained a mouse with constitutive loss of Etnppl (Etnppl^KO^). These mice are viable and fertile with no overt morphological or behavioral abnormalities. We confirmed that Etnppl protein was lost in the brain and liver *via* western blotting (Fig. 4, *A* and *B*). Since PEtN feeds into a number of complex lipid pathways, loss of Etnppl may alter gene expression of PEtN-related genes as they compensate for the loss of Etnppl activity in both the brain and liver. To investigate this, we performed qRT-PCR using the cortical tissue and liver from 9-week-old wild-type and Etnppl^KO^ mice after an 18-h overnight fast. An assortment of genes relevant to PEtN-related genes (*i.e.*, *Etnk1*, *Etnk2*, *Ect*, *Cept1*, *Pisd*, *Pemt*, Phospho1, etc.), lipid metabolism and regulation genes (*i.e.*, *Ppar⍺*, *Bdh*, *Atgl*, *Cd36*, *Cpt1a*, *Cpt2*, *Fasn*, *Mcad*, *Lcad*, etc.), and genes that are indicators of CNS inflammation and oxidative stress (*i.e.*, *Gfap* and *Sod1*). Interestingly in the brain, loss of Etnppl did not result in any statistically significant changes in mRNA expression of any investigated genes (Fig. 4*C*). In the liver, mRNA expression due loss of Etnppl was significant for multiple genes. *Sod1* liver mRNA expression was increased 1.5-fold and *Aldh1l1* was increased 7.6-fold in Etnppl^KO^ compared with wild-type liver (Fig. 4*D*). FAO gene *Hadh* was increased 3.1-fold in Etnppl^KO^ compared with WT liver (Fig. 4*D*). Interestingly loss of Etnppl only resulted in significant expression changes in the liver. Perhaps differences in lipid composition, abundances of critical lipids between the brain and liver, or redundant roles of enzymes found in the brain account for the mRNA expression changes due to Etnppl loss found in the liver but not in the brain.

### Fatty acid oxidation (FAO) and incorporation of ethanolamine (EtN) into membrane lipids in the brain are unchanged due to loss of Etnppl

The substrate that Etnppl irreversibly degrades, PEtN, is commonly found as a head group in the abundant membrane phospholipid PE. PEtN as a phospholipid head group is critical for maintaining proper membrane phospholipid curvature (50). Dysregulation of PEtN may have an impact on neurologic function by perturbing PE biosynthesis and as a result membrane structure. Furthermore, previous studies have identified that PE is essential for neural development and for proper myelination (16, 51). It is expected that if *Etnppl* expression is decreased or Etnppl protein is lost, then there would be greater available PEtN to be incorporated into PE. To determine the impact of Etnppl loss on the incorporation of PEtN into PE, we examined the incorporation of ^14^C-ethanolamine into complex lipids from postnatal day 2 1° cortical astrocytes from wild-type and constitutive Etnppl^KO^ brains (52). Wild-type and Etnppl^KO^ astrocytes were treated with DMSO vehicle control or 100 nM dexamethasone for 24-h followed by a 4-h incubation with 1-^14^C-ethanolamine. There were no significant changes in incorporation of radiolabeled ethanolamine due to Etnppl loss or dexamethasone treatment (Fig. 4*E*). This suggests that the loss of Etnppl is not sufficient to alter incorporation of EtN into PE.

PEtN, the metabolite acted upon by Etnppl, was previously shown to inhibit mitochondrial respiration *via* an unknown mechanism (29). To address the consequences of Etnppl loss on oxidative metabolism in the brain, oxidation of the fatty acid oleate was measured by capturing radiolabeled ^14^CO_2_ from consumed 1-^14^C-oleate using 1° P2 cortical astrocytes harvested from wild-type and constitutive Etnppl^KO^ brains. The FAO inhibitor etomoxir was used as a control of FAO loss using the wild-type 1° astrocytes (Fig. 4*F*). There were no observed or statistically significant changes in ^14^C-oleate consumption in Etnppl^KO^ astrocytes.

**Figure 5:**
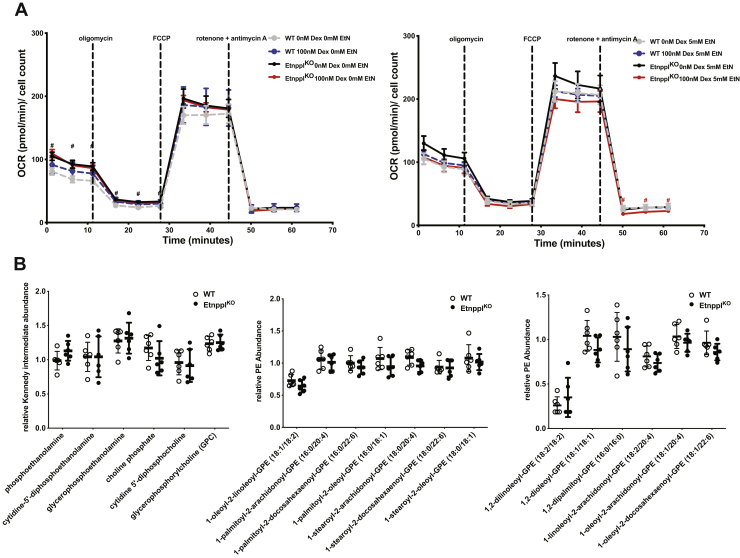
**Loss of Etnppl does not result in major changes to oxygen consumption or abundance of many hippocampal PEtN-related metabolites.***A*, seahorse assay mitochondrial stress test measuring oxygen consumption using cultured P2 1° cortical astrocytes derived from Etnppl^KO^ and WT mice after overnight incubation with dexamethasone and ethanolamine (EtN) (n = 6). [dexamethasone] = 100 nM, [EtN] = 5 mM. *B*, relative abundances of PEtN-associated metabolites in whole hippocampus from 18-h fasted 9-week-old Etnppl^KO^ and WT (n = 6). Data in A are expressed as mean ± S.E.M. Represented data analyzed using multiple Student’s two-tailed t-tests. Statistical significance of represented metabolites in B determined using two-stage false discovery rate (FDR) method of Benjamini, Krieger, and Yekutieli with an FDR (Q) of 10%. Fold changes in green boxes are significantly increased, fold changes in red boxes are significantly decreased, and fold changes in yellow boxes are not significantly affected by genotype. The same data are represented as mean of relative species abundance ± S.D. in adjacent graphs in panels *C*–*E*. ∗α = 0.05; ∗∗α = 0.01; ∗∗∗α = 0.001; ∗∗∗∗α = 0.0001; ns, not significant.

Loss of Etnppl may impact the oxidation of metabolic substrates other than fatty acids. To more widely assess the impact of Etnppl on oxidative capacity, a mitochondrial stress test, which reads real time oxygen consumption, was performed using the Agilent Seahorse platform using wild-type and Etnppl^KO^ P2 1° cortical astrocytes. Astrocytes were incubated with either 100 nM dexamethasone or DMSO vehicle control as well as simultaneously incubated with either 0 mM or 5 mM ethanolamine as substrate for Etnppl protein, for a total of four groups, for 24-h prior to analysis in the Seahorse flux analyzer. Over the course of the mitochondrial stress test, a series of electron transport chain inhibitors were used to assess mitochondrial health and oxidative capacity. Oxygen consumption in the Seahorse platform was significantly altered by genotype at particular parts of the oxygen consumption trace under certain EtN and dexamethasone conditions. At baseline, before injection of any inhibitors, basal oxygen consumption was approximately 1.35-fold higher in Etnppl ^KO^ astrocytes than in WT astrocytes when both had no added EtN or dexamethasone (Fig. 5*A*). Oxygen consumption in Etnppl^KO^ astrocytes, in the absence of dexamethasone, was also significantly increased after oligomycin indicating a minor increase in proton leak (Fig. 5*A*). Oxygen consumption was 1.37-fold lower in Etnppl^KO^ astrocytes, compared with wild-type astrocytes, after inhibition of OXPHOS using rotenone and antimycin A in cells that had been incubated with 100 nM dexamethasone and 5 mM ethanolamine indicating a lower extent of nonmitochondrial oxygen consumption in Etnppl^KO^ astrocytes (Fig. 5*A*). Overall, none of these significant differences were robust, yet they could indicate a subtle decreased capacity for substrate oxidation.

Utilizing global unbiased metabolomics, we determined the relative abundances of 568 metabolites including Kennedy pathway intermediates, various phosphatidylethanolamine species, various phosphatidylcholine species, species from several other classes of phospholipids, as well as many other lipids, amino acids, and carbohydrates that are less relevant to Etnppl and PEtN metabolism. Overwhelmingly, very few metabolites in the overnight fasted 9-week-old hippocampus were statistically significantly altered due to the loss of Etnppl (Fig. 5*B*, Fig. S4).

### Membrane phospholipids are more abundant in the Etnppl^KO^ cortex

Global metabolomics profiles only provide a limited window of changes in lipids. To address this, we performed targeted lipidomics specifically to examine a comprehensive panel of phospholipids found in analyzed samples. Two sets of samples were used for analysis. First, cortex from 9-week-old overnight, 18-h fasted wild-type and Etnppl^KO^ mice. Cortex samples were analyzed by the Mass Spectrometry Lipidomics Core Facility in the Pharmacology Department at the University Anschutz Medical Campus in Aurora, Colorado, the University of Colorado, Aurora Lipidomics Core for targeted mass spectrometry of the species within the phospholipid classes of PEs and phosphatidylcholines (PCs). Secondly, liver samples were collected from 9-week-old overnight, 18-h fasted wild-type and Etnppl^KO^ mice.

**Figure 6:**
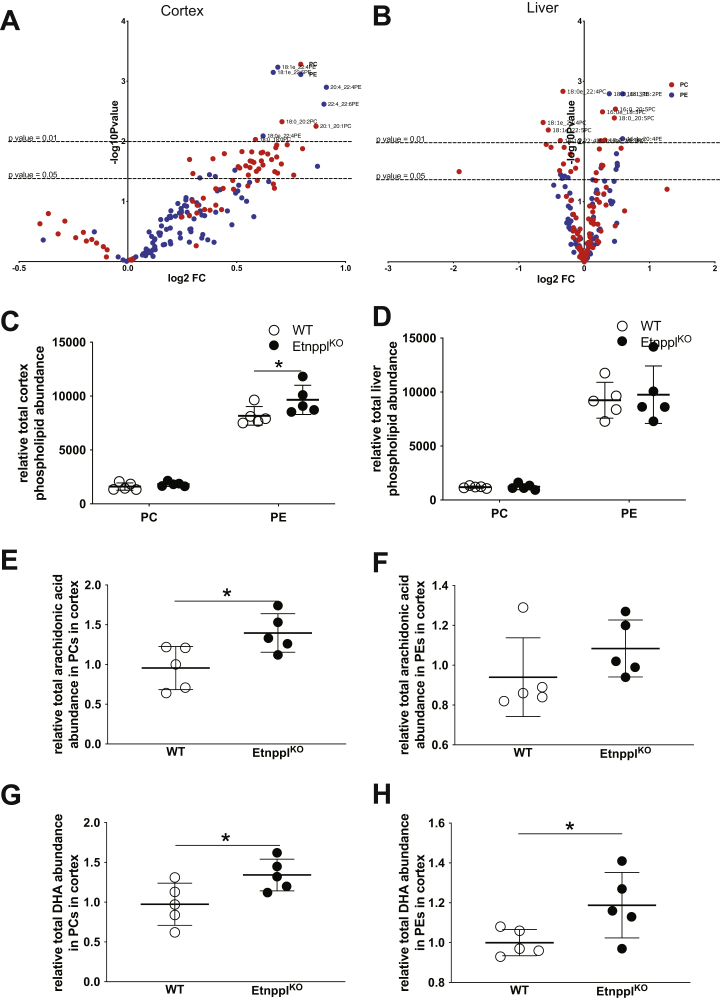
**Phospholipid abundance and composition are altered in cortex after loss of Etnppl.***A*, volcano plot representing PC and PE species fold changes comparing Etnppl^KO^ with WT using 18-h fasted cortex from 9-week-old mice. n = 5. *B*, volcano plot representing phospholipid species fold changes comparing Etnppl ^KO^ with WT using 18-h fasted liver from 9-week-old mice. *C*, total relative phospholipid abundance in the cortex from 18-h fasted, 9-week-old Etnppl^KO^ and WT mice. n = 5. *D*, total relative phospholipid abundance in the cortex from 18-h fasted liver from 9-week-old Etnppl ^KO^ and WT mice. n = 5. *E*, relative total AA abundance in PC species in the cortex from 18-h fasted, 9-week-old Etnppl ^KO^ and WT mice. n = 5. *F*, relative total AA abundance in PE species in the cortex from 18-h fasted, 9-week-old Etnppl^KO^ and WT mice. n = 5. *G*, relative total DHA abundance in PC species in the cortex from 18-h fasted, 9-week-old Etnppl^KO^ and WT mice. n = 5. *H*, relative total DHA abundance in PE species in the cortex from 18-h fasted, 9-week-old Etnppl ^KO^ and WT mice. n = 5. Data are expressed as mean ± S.D. Represented data analyzed using Student’s two-tailed t-tests. ∗α = 0.05; ∗∗α = 0.01; ∗∗∗α = 0.001; ∗∗∗∗α = 0.0001; ns, not significant.

Cortex lipidomics data revealed that the abundances of many individual species of PEs and PC were statistically significantly altered in the cortex by loss of Etnppl. The overwhelming majority of PE species that were statistically significantly changed in cortex samples were modestly increased due to Etnppl loss ranging from 1.9-fold to 1.3-fold. Many of the PEs increased in Etnppl^KO^ cortex were also comprised of at least one long-chain poly- and monounsaturated fatty acids, including arachidonic acid (AA, C20:4n6) and DHA (C22:6n3) (Fig. 6*A*, Fig. S5). Total PC was unchanged but total PE abundance was increased in Etnppl^KO^ compared with wild-type (Fig. 6*C*). Total abundance of DHA in PEs was significantly increased 1.19-fold in Etnppl^KO^ cortex compared with wild-type (Fig. 6*H*). There was a trending increase in total AA in PE from Etnppl^KO^ cortex compared with wild-type (Fig. 6*F*). The latter two fatty acids have a higher abundance in the brain at baseline than in other tissues (4, 53, 54).

While total abundance of PCs was not significantly increased in Etnppl^KO^ cortex, a large number of individual PC species were increased. Observed PC species that were significantly increased in Etnppl^KO^ cortex follow similar trends to those of PE containing at least one PUFA (Fig. 6*A*, Fig. S6). Total abundance of DHA in PCs in Etnppl^KO^ cortex was significantly elevated 1.34-fold compared with wild-type cortex (Fig. 6*G*). Total AA in phosphatidylcholines in Etnppl^KO^ cortex is significantly elevated 1.4-fold compared with wild-type cortex (Fig. 6*E*).

In the liver, overall changes in phospholipid abundance were less impacted due to loss of Etnppl than in the brain (Fig. 6*B*). Total abundance was not significantly changed in PC or PE in the liver samples as in the cortex (Fig. 6*D*). However, similar to the cortex, many individual phospholipids species were significantly increased due to the loss of Etnppl in the liver. While none of the PC species were significantly decreased in Etnppl^KO^ cortex, in Etnppl^KO^ liver, several PCs were increased and several decreased (Fig. 6*B*). In the brain, we observed that the loss of Etnppl increases the overall abundance of PEs and most dramatically increases the abundance of brain PEs containing PUFAs such as DHA and AA (Fig. 6). This suggests that Etnppl is critical in the maintenance of brain phospholipid homeostasis especially comprised of PUFAs with high neurologic relevance. In the liver, while overall abundance of either phospholipid class was not significantly changed; however, the changes in specific phospholipids follow a similar trend as in the brain. Overwhelmingly, phospholipids species that contain PUFAs significantly elevated suggesting that the Etnppl may be responsible for regulating phospholipid acyl-chain homeostasis.

## Discussion

*Etnppl* expression has been observed to be altered throughout *in vivo* studies using rodent models and in human clinical studies in a number of contexts including the following: mood disorders (34, 37), social stress (48, 49), and cancers (38, 39) in multiple tissues. Here, we have characterized the regulation and physiological role of Etnppl *in vivo*. Etnppl sits at the cornerstone of several species of complex lipids, and these data evaluate dietary fasting (Fig. 1, *B*, *C* and *E*) as an additional modulator of Etnppl expression. While these data support others’ findings and a role for Etnppl in maintaining phospholipid homeostasis, there is still much to learn regarding the relevance and function(s) of Etnppl both in the mammalian CNS and throughout peripheral tissues. Our data suggests that Etnppl is critical for maintaining phospholipid homeostasis, which is known to have an impact on neurologic function (15, 16, 18, 55).

*Etnppl* expression increases dramatically between the second and third weeks of postnatal life in the brain. Major changes in lipid metabolism occur in mammals over the course of development. Elevated utilization of lipids for versatile roles such as incorporation into membrane lipids and myelin as well as for elevated fatty acid utilization coincides with major periods of brain growth. In humans, the brain at birth is approximately 27% of its adult weight, which quickly increases to approximately 80% of its adult weight by 2 years of age (56, 57). Much of this new growth includes lipids, which comprise 50% of total adult dry brain mass (58). In rodents, there is evidence of increased fatty acid oxidation in early postnatal development between 15 and 30 days of age (approximately around weaning age), which also correlates to a major period of rodent glial development (59, 60). Myelination in the rodent CNS increases 500% within this developmental window and astrocytes, which overwhelmingly express Etnppl within the brain, are capable of regulating and modulating myelin in the CNS (59, 60, 61). Major increases in brain *Etnppl* mRNA and protein expression determined in our study coincide with this developmental window. Therefore, Etnppl may play a critical developmental role in early neurologic development that has yet to be uncovered.

*Etnppl* expression is regulated by glucocorticoids and in CNS astrocytes following fasting. *In vitro*, when exposed to the glucocorticoid receptor agonist dexamethasone (100 nM) over a time course between 0.25 h and 24 h, astrocytic *Etnppl* mRNA expression was elevated up to 3.72-fold *via* microarray (62). This has been corroborated by our 24-h and 72-h 100 nM dexamethasone-treated P2 1° astrocytes. These data also show changes to the Kennedy pathway for PE biogenesis including significant upregulation of the rate-limiting gene *Pcyt2* following dexamethasone exposure, suggesting a larger role for glucocorticoid signaling in regulating PEs (62). In humans, mood disorders such as schizophrenia and bipolar disorder are associated with elevations in stress hormones due to substantial trauma or chronic stress in early development, which also have substantially increased *Etnppl* expression (34, 35, 37, 63). Furthermore, there is evidence that dietary fasting and chronic high-fat diet increase glucocorticoids and alter the abundance of glucocorticoid receptors through altered rhythmicity of the hypothalamic–pituitary–adrenocortical (HPA) axis (64). Glucocorticoid signaling is already known to impact systemic lipid metabolism distinctly in different tissues. Overall, glucocorticoid signaling results in downstream physiological responses that ultimately are meant to generate a sustained glucose supply for the brain while simultaneously decreasing glucose uptake in the peripheral tissues and elevating circulating fatty acids. For example, glucocorticoids stimulate hepatic lipogenesis and triacylglycerol synthesis while promoting lipolysis in the white adipose tissue (65).

While glucocorticoid signaling may stimulate expression of *Etnppl*, a neurologic or systemic functional role for Etnppl remains undetermined. The known catalytic role of Etnppl is to irreversibly break down PEtN into acetaldehyde, ammonium, and inorganic phosphate (24). PEtN is an amino acid that is a component of complex structural lipids including PE (27), sphingosine-1-phosphate (42), certain plasmalogens (16) and endocannabinoids (43, 44), and as a potential inhibitor of mitochondrial respiration (29). In the brain, the composition and abundance of lipids are unique compared with other tissues to accomplish complex neurologic signaling and to maintain proper neurologic structure (66, 67). One major structural component that contributes largely to unique brain lipid composition is the lipid-laden myelin (68). It has been previously determined that myelin sheath thickness is plastic and is modulated by perinodal astrocytes as a means to impact conduction velocity (69). This is evidence that astrocytes, which express the majority of Etnppl in the brain, modulate the lipid structure within the nervous system. Astrocytes have previously been characterized as important in other regards of brain lipid metabolism including being the main site of fatty acid oxidation (70) and cholesterol synthesis (71) within the CNS. Perhaps, induction of Etnppl occurs in astrocytes after a period of food insecurity to regulate PEtN or a PEtN-containing complex lipid such as PE as a means of protection for the CNS.

Modulation of PEtN and EtN abundance has been previously described to have an impact on neurologic function. Mutant eas flies are unable to generate the critical intermediate PEtN and have an altered membrane lipid composition. These neurologic deficits can be rescued in *Drosophila* by the administration of exogenous PEtN (31). Furthermore, there is evidence that administration of exogenous PEtN prevents autoimmune encephalitis when administered *via* intraperitoneal (i.p.) injections in rats (72). Etnppl protein has been found to have decreased expression in human glioma brain tumors, which further decreases with malignant progression (38). Since PEtN is a critical component of many complex lipids, most notably PE, it is speculated that decreased Etnppl and therefore increased PEtN abundance found in multiple cancers would allow for greater unchecked cellular growth, common to cancers. However, the normal functional role of Etnppl within the nervous system remains uncharacterized. What we can gather is that the main substrate for Etnppl, PEtN, is critical for proper neurologic function based on its previously determined therapeutic benefit. Previous studies have observed Etnppl expression both in the cytosol and within the nucleus (38, 73). Perhaps Etnppl is necessary for regulating the concentration of PEtN throughout multiple cellular compartments, or it could be the case that one of the by-products of PEtN degradation, such as acetaldehyde, ammonium, or inorganic phosphate plays a signaling role in the glia, which is regulated by Etnppl.

Etnppl is known to be elevated in several neurologic disorders including schizophrenia and bipolar disorder while being decreased in depression (34, 37). Our group has determined that *Etnppl* is regulated by dietary fasting specifically in astrocytes within the brain (and liver). Using cultured P2 1° astrocytes, we validated previous findings that Etnppl is also induced by glucocorticoids. We determined that mRNA and protein expression of Etnppl increase dramatically in early postnatal development between the second and fourth weeks of life in mouse brain and liver. Furthermore, loss of the PEtN phospholyase, Etnppl results in elevations of PE abundance to the hippocampal lipidome, which could be attributed to elevated PEtN to incorporate into PEs or altered turnover rates of all phospholipids. No major changes in overt morphology, gene expression, or substrate utilization were observed due to the loss of Etnppl. However, there is a possibility that PHOSPHO1, which has similar activity as Etnppl, is functionally redundant or that developmental compensation exists using a mouse model with a constitutive loss of Etnppl. It is possible that an animal model with inducible loss of Etnppl or an overexpression model of Etnppl, to overcome the potentially redundant Phospho1, would result in a more robust impact/impairment to neurologic function and the brain metabolome. While there is still much undetermined regarding any functional roles for Etnppl, we have demonstrated its means of dietary and developmental regulation and its impact on the brain lipid metabolome.

## Experimantal procedures

### Animals

All procedures were performed in accordance with the *NIH’s Guide for the Care and Use for Laboratory Animals* and under the approval of the Johns Hopkins Medical School Animal Care and Use Committee.

Constitutive Etnppl^KO^ mice were acquired from Taconic Biosciences (#10062). Mice were housed in ventilated racks with a 14-hr-light/10-hr-dark cycle and fed a standard chow diet (2018SX, Teklad Global). Fed and 24-h fasted mice were euthanized at the same time of day (9 AM) at 9 weeks of age unless otherwise noted. For fasting studies, mice were deprived of food for 18 h (3 PM–9 AM). Food deprivation schedule and timing of tissue collection were consistent.

### Translating ribosomal affinity purification

Actively translating mRNAs were determined using translating ribosomal affinity purification (TRAP) based on established protocols (40, 74, 75). Ribo-Tag transgenic mouse expression of pull-down epitope was under the control of neuron-specific synapsin-Cre or astrocyte-specific Aldh1l1-Cre. Neuron and astrocyte specific Ribo-Tag mice were subjected to a normal chow diet (2018SX, Teklad Global), ketogenic diet (41), or fasted overnight for 18 h prior to dissection and tissue harvest. Ribosomes were pulled down and mRNA was isolated from hippocampus. mRNA expression was determined using Affymetrix mouse whole exome microarray and validated by qRT-PCR.

### Primary (1°) astrocyte isolation and culture

T-25 culture flasks (Falcon 353109) (one per cortex) were coated with 1:500 rat tail collagen I (Thermo Fisher A1048301) in Dulbecco’s modified eagle medium (DMEM) for 4 h. Afterward, culture flasks were washed with 1X phosphate-buffered saline (PBS) (Quality Biological) and set to dry. Brains from postnatal day 2 (P2) CPT2^lox/lox^ and CPT2^B−/−^ mice were rapidly dissected in ice-cold 1X Hank’s buffered salt solution (HBSS) (no Ca^2+^, no Mg^2+^, Thermo Fisher). Hippocampus, midbrain, cerebellum, and brainstem were discarded from each cortex and meninges were completely removed. On ice, cortex tissue 1X HBSS was gently minced using sterile razor blades. Minced cortex and 4 ml of HBSS were recovered and transferred to 14 ml round-bottom culture tubes. One milliliter of 0.25% trypsin-EDTA was added to each round-bottom tube and was gently shaken for 25 min at 37 °C. Cortices were washed twice with DMEM, 15% fetal bovine serum, and 1% penicillin/streptomycin antibiotic (Invitrogen) twice to remove Trypsin-EDTA (Thermo Fisher). Tissues were dissociated by gently triturating using a 2 ml sterile serological pipette followed by three subsequent triturations using a sterile Pasteur pipette. Supernatant from each round of triturations was transferred to a new round-bottom tube. Cell suspension was passed through a 40 μM cell strainer and resuspended in 5 ml of DMEM, 15% fetal bovine serum, and 1% penicillin/streptomycin antibiotic (Invitrogen) on coated T-25 flasks. Media was changed every 2 to 3 days. Astroglia grew to confluency within 10 to 14 days.

### Fatty acid oxidation, glucose uptake, and ethanolamine (EtN) incorporation

All labeling experiments were performed using P2 1° cortical astrocytes from wild-type and Etnppl^KO^ pups seeded in 1:500 rat collagen (ThermoFisher A1048301) coated T-25 flasks or 6-well culture dishes 48-h prior to the experiment. All assays were conducted between passages 2 and 4. For FAO experiments [protocol modified from Jernberg *et al.*, 2017 (76)], 200,000 astrocytes per flask were labeled in stoppered T-25 flasks with labeling media containing 0.12 μCi [1-^14^C] oleic acid (Moravek Biochemicals) and incubated at 37 °C (5% CO_2_, 90% relative humidity) for 4 h. Labeling media was composed of 20% Neurobasal medium (Gibco; 21103-049) and 80% glucose-, glutamine-, and pyruvate-free DMEM (Gibco; A14430) supplemented such that final concentrations were 5 mM glucose, 25 μM glutamine, 50 μM sodium pyruvate, and 0.2 mM carnitine, and 0.1% (w/v) bovine serum albumin (Sigma A9647). Etomoxir-treated samples were incubated 100 μM etomoxir (Sigma E1905) in the labeling medium administered at the time of initiating the assay. ^14^CO_2_ was trapped on Whatman filter paper suspended in the headspace of the flask using a center well by addition of 200 μl of 70% perchloric acid in the media and 150 μl of 1 M NaOH directly on the filter paper and incubated at 55 °C for 1 h. The filter paper was placed in 4 ml of scintillation fluid and radioactivity was measured. Astrocytes were lysed using 0.5 ml 1X Triton-X-100 in 1X PBS. Counts were normalized to total microgram protein determined by bicinchoninic acid (BCA) assay.

Ethanolamine incorporation was measured using 100,000 astrocytes per well in 6-well dishes with labeling media containing 0.1 μCi/well [1-^14^C] ethanolamine (Moravek Biochemicals) and incubated at 37 °C (5% CO_2_, 90% relative humidity) for 4 h. Labeling media was composed of 20% Neurobasal medium (Gibco; 21103-049) and 80% glucose-, glutamine-, and pyruvate-free DMEM (Gibco; A14430) supplemented such that final concentrations were 5 mM glucose, 25 μM glutamine, and 50 μM sodium pyruvate. Each culture was thoroughly washed in ice-cold 1X PBS. Folch organic extractions were accomplished with additions of 700 μl of 2:1 chloroform methanol with immediate subsequent additions of 450 μl 2 mM magnesium chloride (MgCl_2_) (52). After vortexing and discarding the aqueous layer, the organic layer is “washed” by the addition of 450 μl 2:1 CHCl_3_:MeOH and 300 μl MgCl_2_. The aqueous layer was completely removed and 400 μl of the organic phase was added to each scintillation tube and counted in 4 ml of scintillation fluid and normalized to total microgram protein determined by BCA assay.

### Unbiased global metabolomics

Global metabolomics were performed on rapidly dissected and frozen (in liquid nitrogen) hippocampus from 18-h fasted 9-week-old mice (3 PM–9 AM) as described previously (70, 77, 78, 79, 80).

### Lipidomics

Lipidomics were performed by established methods by the Lipidomics and Mass Spectrometry Core in the Pharmacology Department of the University of Colorado, Anschutz Medical Campus in Aurora, CO (81).

### RNA isolation, purification, and qRT-PCR

RNA were isolated from brain tissues using TRIzol (Life Technologies) and further purified using RNeasy Mini Kit (Qiagen). RNA was quantified by a NanoDrop spectrophotometer (Thermo Fisher Scientific), and cDNA was synthesized using 0.5 to 2 μg of total RNA, random primers, and MultiScribe High-Capacity cDNA reverse transcription kit according to manufacturer’s instructions (Cat no. 4368814; Life Technologies). qRT-PCR was performed using 10 ng of template cDNA and Bio-Rad SsoAdvanced Universal SYBR Green master mix (Cat no. 1725274) with primers specific to the genes of interest. PCR reactions were carried out in a Bio-Rad CFX Connect thermocycler [95 °C for 10 s, 56–95 °C at 0.5 °C/5 s]. All data were normalized to average of housekeeping Ct values from Rpl22 and 18S. Normalized data were expressed as 2^−ΔCt^.

### Statistical analysis

Data were analyzed using Prism 7.0 software (GraphPad). Statistical significance of data was determined using unpaired Student’s two-tailed t-tests for single variable experiments. For unmatched multiple-variable experiments, ordinary two-way analysis of variance (ANOVA) with Šídák corrections for multiple comparisons was used to determine statistical significance of data. For paired multiple-variable experiments, repeated measures two-way ANOVA with Šídák corrections for multiple comparisons were used to determine statistical significance of data.

## Data availability

Microarray data has been deposited in Gene Expression Omnibus GSE169625.

## Supporting information

This article contains supporting information.

## Conflict of interest

The authors have no competing financial interests.

## References

[1] Davis-Bruno K., Tassinari M.S. (2011). Essential fatty acid supplementation of DHA and ARA and effects on neurodevelopment across animal species: A review of the literature. Birth Defects Res. B Dev. Reprod. Toxicol..

[2] Harvey L.D., Yin Y., Attarwala I.Y., Begum G., Deng J., Yan H.Q., Dixon C.E., Sun D. (2015). Administration of DHA reduces endoplasmic reticulum stress-associated inflammation and alters microglial or macrophage activation in traumatic brain injury. ASN Neuro.

[3] Lin L.E., Chen C.T., Hildebrand K.D., Liu Z., Hopperton K.E., Bazinet R.P. (2015). Chronic dietary n-6 PUFA deprivation leads to conservation of arachidonic acid and more rapid loss of DHA in rat brain phospholipids. J. Lipid Res..

[4] Brenna J.T., Diau G.Y. (2007). The influence of dietary docosahexaenoic acid and arachidonic acid on central nervous system polyunsaturated fatty acid composition. Prostaglandins Leukot. Essent. Fatty Acids.

[5] Brenna J.T., Carlson S.E. (2014). Docosahexaenoic acid and human brain development: Evidence that a dietary supply is needed for optimal development. J. Hum. Evol..

[6] Ahmed A.T., MahmoudianDehkordi S., Bhattacharyya S., Arnold M., Liu D., Neavin D., Moseley M.A., Thompson J.W., Williams L.S.J., Louie G., Skime M.K., Wang L., Riva-Posse P., McDonald W.M., Bobo W. (2020). Acylcarnitine metabolomic profiles inform clinically-defined major depressive phenotypes. J. Affect. Disord..

[7] Barone R., Alaimo S., Messina M., Pulvirenti A., Bastin J., MIMIC-Autism Group, Ferro A., Frye R.E., Rizzo R. (2018). A subset of patients with autism spectrum disorders show a distinctive metabolic profile by dried blood spot analyses. Front. Psychiatry.

[8] Clark-Taylor T., Clark-Taylor B.E. (2004). Is autism a disorder of fatty acid metabolism? Possible dysfunction of mitochondrial β-oxidation by long chain acyl-CoA dehydrogenase. Med. Hypotheses.

[9] Ferguson J.N., Young L.J., Insel T.R. (2002). The neuroendocrine basis of social recognition. Front. Neuroendocrinol..

[10] Rossignol D.A., Frye R.E. (2011). Mitochondrial dysfunction in autism spectrum disorders: A systematic review and meta-analysis. Mol. Psychiatry.

[11] Xie Z., Jones A., Deeney J.T., Hur S.K., Bankaitis V.A. (2016). Inborn errors of long-chain fatty acid β-oxidation link neural stem cell self-renewal to autism. Cell Rep..

[12] Merritt J.L., Norris M., Kanungo S. (2018). Fatty acid oxidation disorders. Ann. Transl. Med..

[13] Tyni T., Pihko H. (1999). Long-chain 3-hydroxyacyl-CoA dehydrogenase deficiency. Acta Paediatr..

[14] Tyni T., Palotie A., Viinikka L., Valanne L., Salo M.K., von Döbeln U., Jackson S., Wanders R., Venizelos N., Pihko H. (1996). Long-chain 3-hydroxyacyl-coenzyme A dehydrogenase deficiency with the G1528C mutation: Clinical presentation of thirteen patients. J. Pediatr..

[15] Schuurs-Hoeijmakers J.H., Geraghty M.T., Kamsteeg E.J., Ben-Salem S., de Bot S.T., Nijhof B., van de Vondervoort I.I., van der Graaf M., Nobau A.C., Otte-Höller I., Vermeer S., Smith A.C., Humphreys P., Schwartzentruber J., FORGE Canada Consortium (2012). Mutations in DDHD2, encoding an intracellular phospholipase A1, cause a recessive form of complex hereditary spastic paraplegia. Am. J. Hum. Genet..

[16] Vaz F.M., McDermott J.H., Alders M., Wortmann S.B., Kölker S., Pras-Raves M.L., Vervaart M.A.T., van Lenthe H., Luyf A.C.M., Elfrink H.L., Metcalfe K., Cuvertino S., Clayton P.E., Yarwood R., Lowe M.P. (2019). Mutations in PCYT2 disrupt etherlipid biosynthesis and cause a complex hereditary spastic paraplegia. Brain.

[17] Ahmed M.Y., Al-Khayat A., Al-Murshedi F., Al-Futaisi A., Chioza B.A., Pedro Fernandez-Murray J., Self J.E., Salter C.G., Harlalka G.V., Rawlins L.E., Al-Zuhaibi S., Al-Azri F., Al-Rashdi F., Cazenave-Gassiot A., Wenk M.R. (2017). A mutation of EPT1 (SELENOI) underlies a new disorder of Kennedy pathway phospholipid biosynthesis. Brain.

[18] Lamari F., Mochel F., Saudubray J.M. (2014). An overview of inborn errors of complex lipid biosynthesis and remodelling. J. Inherit. Metab. Dis..

[19] Cho K.H., Shim S.H., Kim M. (2017). Clinical, biochemical, and genetic aspects of Sjögren-Larsson syndrome. Clin. Genet..

[20] Huigen M.C., van der Graaf M., Morava E., Dassel A.C., van Steensel M.A., Seyger M.M., Wevers R.A., Willemsen M.A. (2015). Cerebral lipid accumulation in Chanarin–Dorfman syndrome. Mol. Genet. Metab..

[21] Schiroli D., Peracchi A. (2015). A subfamily of PLP-dependent enzymes specialized in handling terminal amines. Biochim. Biophys. Acta.

[22] Schiroli D., Cirrincione S., Donini S., Peracchi A. (2013). Strict reaction and substrate specificity of AGXT2L1, the human O-phosphoethanolamine phospho-lyase. IUBMB Life.

[23] Schiroli D., Ronda L., Peracchi A. (2014). Kinetic characterization of the human O-phosphoethanolamine phospho-lyase reveals unconventional features of this specialized pyridoxal phosphate-dependent lyase. FEBS J..

[24] Veiga-da-Cunha M., Hadi F., Balligand T., Stroobant V., Van Schaftingen E. (2012). Molecular identification of hydroxylysine kinase and of ammoniophospholyases acting on 5-phosphohydroxy-L-lysine and phosphoethanolamine. J. Biol. Chem..

[25] Fleshood H.L., Pitot H.C. (2016). The metabolism of O-phosphorylethanolamine in animal tissues. II. Metabolic regulation of O-phosphorylethanolamine phospho-lyase in vivo. Arch. Biochem. Biophys..

[26] Pavlovic Z., Bakovic M. (2013). Regulation of phosphatidylethanolamine homeostasis—the critical role of CTP:phosphoethanolamine cytidylyltransferase (Pcyt2). Int. J. Mol. Sci..

[27] Kennedy E.P., Weiss S.B. (1956). The function of cytidine coenzymes in the biosynthesis of phospholipides. J. Biol. Chem..

[28] Dowhan W. (1997). Molecular basis for membrane phospholipid diversity: Why are there so many lipids?. Annu. Rev. Biochem..

[29] Gohil V.M., Zhu L., Baker C.D., Cracan V., Yaseen A., Jain M., Clish C.B., Brookes P.S., Bakovic M., Mootha V.K. (2013). Meclizine inhibits mitochondrial respiration through direct targeting of cytosolic phosphoethanolamine metabolism. J. Biol. Chem..

[30] Pavlidis P., Ramaswami M., Tanouye M.A. (2003). The Drosophila easily shocked gene: A mutation in a phospholipid synthetic pathway causes seizure, neuronal failure, and paralysis. Cell.

[31] Kroll J.R., Tanouye M.A. (2013). Rescue of easily shocked mutant seizure sensitivity in Drosophila adults. J. Comp. Neurol..

[32] Zhang Y., Chen K., Sloan S.A., Bennett M.L., Scholze A.R., O'Keeffe S., Phatnani H.P., Guarnieri P., Caneda C., Ruderisch N., Deng S., Liddelow S.A., Zhang C., Daneman R., Maniatis T. (2014). An RNA-sequencing transcriptome and splicing database of glia, neurons, and vascular cells of the cerebral cortex. J. Neurosci..

[33] Clarke L.E., Liddelow S.A., Chakraborty C., Münch A.E., Heiman M., Barres B.A. (2018). Normal aging induces A1-like astrocyte reactivity. Proc. Natl. Acad. Sci. U. S. A..

[34] Shao L., Vawter M.P. (2008). Shared gene expression alterations in schizophrenia and bipolar disorder. Biol. Psychiatry.

[35] Sibille E., Arango V., Galfalvy H.C., Pavlidis P., Erraji-Benchekroun L., Ellis S.P., John Mann J. (2003). Gene expression profiling of depression and suicide in human prefrontal cortex. Neuropsychopharmacology.

[36] Schroeder F.A., Lewis M.C., Fass D.M., Wagner F.F., Zhang Y.L., Hennig K.M., Gale J., Zhao W.N., Reis S., Barker D.D., Berry-Scott E., Kim S.W., Clore E.L., Hooker J.M., Holson E.B. (2013). A selective HDAC 1/2 inhibitor modulates chromatin and gene expression in brain and alters mouse behavior in two mood-related tests. PLoS One.

[37] McQuillin A., Rizig M., Gurling H.M. (2007). A microarray gene expression study of the molecular pharmacology of lithium carbonate on mouse brain mRNA to understand the neurobiology of mood stabilization and treatment of bipolar affective disorder. Pharmacogenet. Genomics.

[38] Leventoux N., Augustus M., Azar S., Riquier S., Villemin J.P., Guelfi S., Falha L., Bauchet L., Gozé C., Ritchie W., Commes T., Duffau H., Rigau V., Hugnot J.P. (2020). Transformation foci in IDH1-mutated gliomas show STAT3 phosphorylation and downregulate the metabolic enzyme ETNPPL, a negative regulator of glioma growth. Sci. Rep..

[39] Ding Q., Kang J., Dai J., Tang M., Wang Q., Zhang H., Guo W., Sun R., Yu H. (2016). AGXT2L1 is down-regulated in heptocellular carcinoma and associated with abnormal lipogenesis. J. Clin. Pathol..

[40] Heiman M., Kulicke R., Fenster R.J., Greengard P., Heintz N. (2014). Cell type–specific mRNA purification by translating ribosome affinity purification (TRAP). Nat. Protoc..

[41] Ellis J.M., Bowman C.E., Wolfgang M.J. (2015). Metabolic and tissue-specific regulation of acyl-CoA metabolism. PLoS One.

[42] Borowsky A.D., Bandhuvula P., Kumar A., Yoshinaga Y., Nefedov M., Fong L.G., Zhang M., Baridon B., Dillard L., de Jong P., Young S.G., West D.B., Saba J.D. (2012). Sphingosine-1-phosphate lyase expression in embryonic and adult murine tissues. J. Lipid Res..

[43] Tsuboi K., Takezaki N., Ueda N. (2007). The N-acylethanolamine-hydrolyzing acid amidase (NAAA). Chem. Biodivers.

[44] Leishman E., Mackie K., Luquet S., Bradshaw H.B. (2016). Lipidomics profile of a NAPE-PLD KO mouse provides evidence of a broader role of this enzyme in lipid metabolism in the brain. Biochim. Biophys. Acta.

[45] Clarke L.E., Barres B.A. (2013). Emerging roles of astrocytes in neural circuit development. Nat. Rev. Neurosci..

[46] Iannotti F.A., Di Marzo V., Petrosino S. (2016). Endocannabinoids and endocannabinoid-related mediators: Targets, metabolism and role in neurological disorders. Prog. Lipid Res..

[47] Roberts S.J., Stewart A.J., Sadler P.J., Farquharson C. (2004). Human PHOSPHO1 exhibits high specific phosphoethanolamine and phosphocholine phosphatase activities. Biochem. J..

[48] Stankiewicz A.M., Goscik J., Swiergiel A.H., Majewska A., Wieczorek M., Juszczak G.R., Lisowski P. (2016). Social stress increases expression of hemoglobin genes in mouse prefrontal cortex. BMC Neurosci..

[49] Kadmiel M., Cidlowski J.A. (2013). Glucocorticoid receptor signaling in health and disease. Trends Pharmacol. Sci..

[50] McMahon H.T., Boucrot E. (2015). Membrane curvature at a glance. J. Cell Sci..

[51] Horibata Y., Elpeleg O., Eran A., Hirabayashi Y., Savitzki D., Tal G., Mandel H., Sugimoto H. (2018). EPT1 (selenoprotein I) is critical for the neural development and maintenance of plasmalogen in humans. J. Lipid Res..

[52] Folch J., Lees M., Sloane Stanley G.H. (1957). A simple method for the isolation and purification of total lipides from animal tissues. J. Biol. Chem..

[53] Dyall S.C. (2015). Long-chain omega-3 fatty acids and the brain: A review of the independent and shared effects of EPA, DPA and DHA. Front. Aging Neurosci..

[54] Crawford M.A., Sinclair A.J. (2008). Ciba Foundation symposium 3 - lipids, malnutrition & the developing brain. Novartis Found Symposia.

[55] Tasseva G., Bai H.D., Davidescu M., Haromy A., Michelakis E., Vance J.E. (2012). Phosphatidylethanolamine deficiency in mammalian mitochondria impairs oxidative phosphorylation and alters mitochondrial morphology. J. Biol. Chem..

[56] Dobbing J., Sands J. (1973). Quantitative growth and development of human brain. Arch. Dis. Child..

[57] Zheng L., Fleith M., Giuffrida F., O’Neill B.V., Schneider N. (2019). Dietary polar lipids and cognitive development: A narrative review. Adv. Nutr..

[58] Hamilton J.A., Hillard C.J., Spector A.A., Watkins P.A. (2007). Brain uptake and utilization of fatty acids, lipids and lipoproteins: Application to neurological disorders. J. Mol. Neurosci..

[59] Salvati S., Attorri L., Avellino C., Di Biase A., Sanchez M. (2000). Diet, lipids and brain development. Dev. Neurosci..

[60] Norton W.T., Cammer W., Morell P. (1984). Isolation and characterization of myelin. Myelin.

[61] Zuchero J.B., Barres B.A. (2015). Glia in mammalian development and disease. Development.

[62] Carter B.S., Meng F., Thompson R.C. (2012). Glucocorticoid treatment of astrocytes results in temporally dynamic transcriptome regulation and astrocyte-enriched mRNA changes *in vitro*. Physiol. Genomics.

[63] Bennett Ao M.R. (2008). Stress and anxiety in schizophrenia and depression: Glucocorticoids, corticotropin-releasing hormone and synapse regression. Aust. N. Z. J. Psychiatry.

[64] Vasconcelos A.R., Cabral-Costa J.V., Mazucanti C.H., Scavone C., Kawamoto E.M. (2016). The role of steroid hormones in the modulation of neuroinflammation by dietary interventions. Front. Endocrinol. (Lausanne).

[65] Magomedova L., Cummins C.L. (2016). Glucocorticoids and metabolic control. Handb. Exp. Pharmacol..

[66] Mitchell R.W., Hatch G.M. (2011). Fatty acid transport into the brain: Of fatty acid fables and lipid tails. Prostaglandins Leukot. Essent. Fatty Acids.

[67] Watkins P.A., Hamilton J.A., Leaf A., Spector A.A., Moore S.A., Anderson R.E., Moser H.W., Noetzel M.J., Katz R. (2001). Brain uptake and utilization of fatty acids: Applications to peroxisomal biogenesis diseases. J. Mol. Neurosci..

[68] Schmitt S., Castelvetri L.C., Simons M. (2015). Metabolism and functions of lipids in myelin. Biochim. Biophys. Acta.

[69] Dutta D.J., Woo D.H., Lee P.R., Pajevic S., Bukalo O., Huffman W.C., Wake H., Basser P.J., SheikhBahaei S., Lazarevic V., Smith J.C., Fields R.D. (2018). Regulation of myelin structure and conduction velocity by perinodal astrocytes. Proc. Natl. Acad. Sci. U. S. A..

[70] White C.J., Lee J., Choi J., Chu T., Scafidi S., Wolfgang M.J. (2020). Determining the bioenergetic capacity for fatty acid oxidation in the mammalian nervous system. Mol. Cell. Biol..

[71] Ferris H.A., Perry R.J., Moreira G.V., Shulman G.I., Horton J.D., Kahn C.R. (2017). Loss of astrocyte cholesterol synthesis disrupts neuronal function and alters whole-body metabolism. Proc. Natl. Acad. Sci. U. S. A..

[72] Aguado-Llera D., Puebla-Jiménez L., Barrios V., Hernández-Pinto A., Arilla-Ferreiro E. (2011). Role of ethanolamine phosphate in the hippocampus of rats with acute experimental autoimmune encephalomyelitis. Neurochem. Int..

[73] Boukouris A.E., Zervopoulos S.D., Michelakis E.D. (2016). Metabolic enzymes moonlighting in the nucleus: Metabolic regulation of gene transcription. Trends Biochem. Sci..

[74] Heiman M., Schaefer A., Gong S., Peterson J.D., Day M., Ramsey K.E., Suárez-Fariñas M., Schwarz C., Stephan D.A., Surmeier D.J., Greengard P., Heintz N. (2008). A translational profiling approach for the molecular characterization of CNS cell types. Cell.

[75] Doyle J.P., Dougherty J.D., Heiman M., Schmidt E.F., Stevens T.R., Ma G., Bupp S., Shrestha P., Shah R.D., Doughty M.L., Gong S., Greengard P., Heintz N. (2008). Application of a translational profiling approach for the comparative analysis of CNS cell types. Cell.

[76] Jernberg J.N., Bowman C.E., Wolfgang M.J., Scafidi S. (2017). Developmental regulation and localization of carnitine palmitoyltransferases (CPTs) in rat brain. J. Neurochem..

[77] Eckel-Mahan K.L., Patel V.R., Mohney R.P., Vignola K.S., Baldi P., Sassone-Corsi P. (2012). Coordination of the transcriptome and metabolome by the circadian clock. Proc. Natl. Acad. Sci. U. S. A..

[78] Ellis J.M., Wong G.W., Wolfgang M.J. (2013). Acyl coenzyme A thioesterase 7 regulates neuronal fatty acid metabolism to prevent neurotoxicity. Mol. Cell. Biol..

[79] Lee J., Choi J., Scafidi S., Wolfgang M.J. (2016). Hepatic fatty acid oxidation restrains systemic catabolism during starvation. Cell Rep..

[80] Lee J., Wolfgang M.J. (2012). Metabolomic profiling reveals a role for CPT1c in neuronal oxidative metabolism. BMC Biochem..

[81] Murphy R.C., Merrill A.H. (2011). Lipidomics and imaging mass spectrometry. Biochim. Biophys. Acta.

