# Supplementary material for: The role of ethanolamine phosphate phospholyase in regulation of astrocyte lipid homeostasis
Source: J Biol Chem. 2021 May 26;297(1):100830. doi: 10.1016/j.jbc.2021.100830 (PMC8233209; doi:10.1016/j.jbc.2021.100830)
Supplement: Supplemental Figures S1–S6 [file mmc1.pdf]

Supplemental Figure Legends

Figure S1. Schematic of Translating Ribosomal Affinity Purification.

Figure S2. Etnppl expression across tissues. *Etnppl* mRNA expression in wild-type adult liver (liv), kidney (kid), heart, gWAT, iWAT, sol, plant, gut, brain, and BAT. Data (as mentioned in text) are expressed as mean ± S.D. Represented data analyzed using multiple student's two-tailed t-tests. Outliers were removed after using Grubb's outlier test. *α = 0.05; **α = 0.01; ***α = 0.001; ****α = 0.0001; ns, not significant.

Figure S3. Impact of dexamethasone in primary astrocytes on glucocorticoid responsive genes. mRNA expression of glucocorticoid-responsive genes in wild-type P2 1° astrocytes after a 24- or 72-hour exposure the glucocorticoid agonist dexamethasone. [dexamethasone] = 100nM. (n = 3). Data (as mentioned in text) are expressed as mean ± S.D. Represented data analyzed using multiple student's two-tailed t-tests. Outliers were removed after using Grubb's outlier test. *α = 0.05; **α = 0.01; ***α = 0.001; ****α = 0.0001; ns, not significant.

Figure S4. Impact of Etnppl loss on PC metabolites in the hippocampal metabolome. Relative abundances of PC-associated metabolites in whole hippocampus from 18-hour fasted 9-week-old Etnppl^{KO} and WT (n = 6). Data in A are expressed as mean ± S.D. Represented data analyzed using multiple student's two-tailed t-tests. Statistical significance of represented metabolites in B determined using two-stage false discovery rate (FDR) method of Benjamini, Krieger, and Yekutieli with a FDR (Q) of 10%. Fold changes in green boxes are significantly increased, fold changes in red boxes are significantly decreased, and fold changes in yellow boxes are not significantly affected by genotype. *α = 0.05; **α = 0.01; ***α = 0.001; ****α = 0.0001; ns, not significant.

Figure S5. PE abundance and composition is altered in cortex after loss of Etnppl. Relative abundance in PE species in cortex from 18-hour fasted, 9-week-old $Etnppl^{KO}$ and WT mice. (n = 5) Data are expressed as mean ± S.D. Represented data analyzed using multiple student's two-tailed t-tests. *α = 0.05; **α = 0.01; ***α = 0.001; ****α = 0.0001; ns, not significant.

Figure S6. PC abundance and composition is altered in cortex after loss of Etnppl. Relative abundance in PC species in cortex from 18-hour fasted, 9-week-old $Etnppl^{KO}$ and WT mice. (n = 5) Data are expressed as mean ± S.D. Represented data analyzed using multiple student's two-tailed t-tests. *α = 0.05; **α = 0.01; ***α = 0.001; ****α = 0.0001; ns, not significant.
